# ^119^Sn Element-Specific Phonon Density of States of BaSnO_3_

**DOI:** 10.3390/cryst15050440

**Published:** 2025-05-05

**Authors:** Alexey Rulev, Hongxin Wang, Selma Erat, Murat Aycibin, Daniel Rentsch, Vladimir Pomjakushin, Stephen P. Cramer, Qianli Chen, Nobumoto Nagasawa, Yoshitaka Yoda, Artur Braun

**Affiliations:** 1 Laboratory for High Performance Ceramics, Empa. Swiss Federal Laboratories for Materials Science and Technology, Überlandstrasse 129, CH-8600 Dübendorf, Switzerland; 2 SETI Institute, Mountain View, CA 94043, USA; 3 Program of Opticianry, Department of Medical Services and Techniques, Vocational School of Technical Sciences, 33340 Mersin, Türkiye; 4 Department of Nanotechnology and Advanced Materials, Institute of Science, Mersin University, 33340 Mersin, Türkiye; 5 Laboratory for Functional Polymers, Empa. Swiss Federal Laboratories for Materials Science and Technology, Überlandstrasse 129, CH-8600 Dübendorf, Switzerland; 6 Laboratory for Neutron Scattering, Paul Scherrer Institute, CH-5232 Villigen PSI, Switzerland; 7 University of Michigan–Shanghai Jiao Tong University Joint Institute, Shanghai Jiao Tong University, Shanghai 200240, China; 8 Precision Spectroscopy Division, SPring-8/JASRI, Sayo, Hyogo 679-5198, Japan

**Keywords:** NRVS, vibrational spectroscopy, phonon DOS, BaSnO_3_, proton conductor, fuel cell

## Abstract

Vibration spectroscopy is routinely used in analytical chemistry for molecular speciation. Less common is its use in studying the dynamics of reaction and transport processes. A shortcoming of vibration spectroscopies is that they are not inherently specific to chemical elements. Progress in synchrotron radiation-based X-ray technology has developed nuclear resonance vibration spectroscopy (NRVS), which can be used to produce element-specific vibration spectra and partial vibrational density of states (PVDOS), provided the material under investigation contains a Mössbauer-active element. While the method has been recently used successfully for protein spectroscopy, fewer studies have been conducted for condensed matter. We have employed NRVS on the BaSnO_3_ perovskite structure, which is a model compound for ceramic proton conductors in intermediate temperature fuel cells. Since we used ^119^Sn as a Mössbauer isotope, the derived experimental PVDOS is specific to the element Sn in BaSnO_3_. We show how this phonon DOS is used as an experimental anchor for the interpretation of the DFT-calculated PVDOS of BaSnO_3_.

## 1. Introduction

For the chemical analysis of solids, X-ray spectroscopy is a well-known tool which can replace some of the more laborious classical analytical chemistry tools [1]. This also includes the related electron spectroscopy for chemical analyses (ESCA), which requires X-rays to produce surface-sensitive photoelectrons as probes. These probes allow for the detection of chemical elements present in a sample and their molecular speciation, such as oxidation states, types of bonding, and valence band properties, to name a few. Vibration spectroscopy methods, such as infrared and Raman spectroscopy, are optical methods that allow for the determination of molecular states and functional groups. Condensed matter has functionalities which cannot be fully described by their elemental and chemical compositions alone. It has been shown that the vibration properties of solids influence their heat transfer, mass transport, and electric charge transport for electrons, holes, ions, and vacancies (see, for example, [2,3]). These are important material properties for components of electrochemical energy storage and conversion devices like batteries, solar cells, photoelectrochemical cells, fuel cells, and electrolyzers. Phonon modes influence ion transport, for example, in solid-state electrolytes, such as lithium garnets [4] or proton conductors [5].

We have recently investigated proton transport in ceramic proton conductors with a perovskite structure, specifically the Y-substituted barium cerate and zirconate [6,7], and found that the proton transport follows a polaron mechanism, driven by specific vibration modes [5]. This underlines that the phonon density of states (phonon DOS) is a characteristic of materials which should be understood in detail, if the function of the material in the device is to be understood. Infrared (IR) and Raman spectroscopy are limited in that they do not provide element-specific information for the phonon DOS. Progress in X-ray and synchrotron radiation technology has made it possible to carry out nuclear resonance vibration spectroscopy (NRVS), which can produce element-specific vibration spectra and phonon DOS. The method has been applied with great success in protein spectroscopy (in particular to Fe-containing complexes [8–10]) and provides new insight into the dynamics of enzymes in photosynthesis and metabolism. To a lesser extent, solid-state materials have been subjected to this novel method. Lin et al. have determined the phonon DOS of Fe2O3 under high pressure [11].

^57^Fe is the most relevant Mössbauer isotope studied, followed by ^119^Sn. NRVS requires Mössbauer-active isotopes, the number of which is limited in the periodic table of elements and nuclide table. In addition, the preparation and separation of such isotopes are typically expensive. ^119^Sn has a Mössbauer transition at 28.3 keV, and the isotope has been studied well in the past. There exist a number of NRVS studies involving ^119^Sn. Chumakov et al. reported pioneering work as early as 1998 on the NRVS spectrum of a ^119^Sn metal foil, showing fair agreement with the calculated spectrum [12]. Shortly thereafter, they published a rapid communication on the phonon DOS of the b-phase of 119Sn, matching the calculated phonon DOS [13]. They first measured the phonon DOS of ^119^Sn using NRVS (there called nuclear resonant inelastic X-ray scattering, NRIXS) under high pressure, which showed perfect agreement with calculations [14]. A very recent NRVS study on a ^119^Sn-containing compound was carried out by Weinhard and Heske et al. on the solar cell absorber material Cu_2_ZnSn(S_x_,Se_1−x_)_4_—even operando under solar cell operation conditions [15], underlining the progress in the use and diffusion of NRVS resp. NRIXS for compounds used in energy storage and conversion. We have synthesized Ba^119^SnO_3_ and carried out NRVS on the Y-substituted BaSnO_3_ proton conductor and obtained the Sn-projected experimental partial phonon DOS. By comparing it with the computationally derived phonon DOS of BaSnO_3_ and its element-projected calculated phonon components, we are able to derive the partial phonon DOS of the oxygen lattice with greater confidence. This procedure serves as a prelude to further NRVS studies and will provide deeper insight into the role of vibration properties in device functionality.

## 2. Materials and Methods

### 2.1. Synthesis

Barium stannate (BaSnO_3_) was synthesized with a ceramic solid-state method. It is important to note that, due to the need to use a Mössbauer-active element in NRVS, ^119^Sn is a necessary precursor, which is typically available only as a metal sheet. ^119^Sn was obtained as a metal sheet (Neonest AB (BuyIsotope.com), SE-171 52 Solna, Sweden, enriched with ^119^Sn to 96.3% according to their specification).

Tin metal (GoodFellow, 99.999% purity, CAS 7440–31-5), obtained with a natural isotope distribution of 8.59% of ^119^Sn, was used as a reference material to examine the extent of ^119^Sn enrichment in the sample. The ^119^Sn concentration in the metal was confirmed to be 84% by nuclear magnetic resonance (NMR).

For BaSnO3 synthesis, the Sn metals were dissolved in concentrated high-purity nitric acid (Sigma-Aldrich, 70%, purified by redistillation, ≥99.999% trace metals basis) to obtain tin nitrate. Tin nitrate precipitate was obtained by adding high-purity NH_4_OH. The filtrate residual was heated until a dry powder of BaSnO_3_ was obtained, the phase purity of which was confirmed by X-ray diffraction. The tin nitrate was mixed in stoichiometric amounts with BaCO_3_ (Sigma CAS-Nr.: 513–77-9) to obtain BaSnO_3_. The homogeneous mixture was heated in clean zirconia boat crucibles in an oxygen-vented tube furnace at 1500 K for 12 h. The obtained reaction product was collected from the crucibles and compacted to pellets with 0.5 mm thickness and 8 mm diameter at a force of 150 kN. Note that the specimen was not sintered.

### 2.2. Neutron Diffraction

Neutron powder diffractograms were recorded at the HRPT neutron beamline at the Swiss Spallation Neutron Source in Villigen, Switzerland [16–18]. For neutron diffraction, we used powder samples, prepared as described above from natural-abundance isotope tin foil from Goodfellow. Diffractograms were acquired at a neutron wavelength of 1.1545 Å at temperatures of 1, 100, and 200 K. The stoichiometry, as determined by Rietveld refinement of the neutron diffraction data, is shown in Table 1. The refinement was performed with the GSAS-II package [19]. During the refinement, the Ba occupancy was fixed at 1, and the Sn and O occupancies were kept the same across all temperatures. The Sn occupancy was 0.970±0.003 and the O occupancy was 0.975 ± 0.003, so the stoichiometry was BaSn_0.97_O_2.925_. Hence, approx. 1.5% of Sn was Sn^2+^: net charge = 0 = 2.925· (−2) + 1 · (+2) + (0.97−x) · (+4) + x· (+2); x = 0.015. Thus, the nominal BaSnO_3_ contained 2.5% oxygen vacancies, allowing for proton transport even without substituting Sn^4+^ with Y^3+^. These vacancies affect the transport properties, including thermal transport [20].

### 2.3. Nuclear Resonance Vibration Spectroscopy

Nuclear resonance vibration spectroscopy (NRVS) measurements were collected at beamline BL35XU at SPring-8 in Hyogo, Japan [21,22]. Pellets with diameters of 8 mm were measured at 298 K. The irradiated area was 2 mm in diameter. The X-ray energy was set to 23.87 keV to excite the γ-transition of ^119^Sn [23]. Acquisition of one spectrum covering the range from −30 to 120 meV took approximately 24 h. All the measurements were performed at ambient temperature under vacuum. NRVS spectra were then processed using the online “NRVS Tool” from spectra.tools [24] based on the software package of PHOENIX [25].

**Figure 1.**
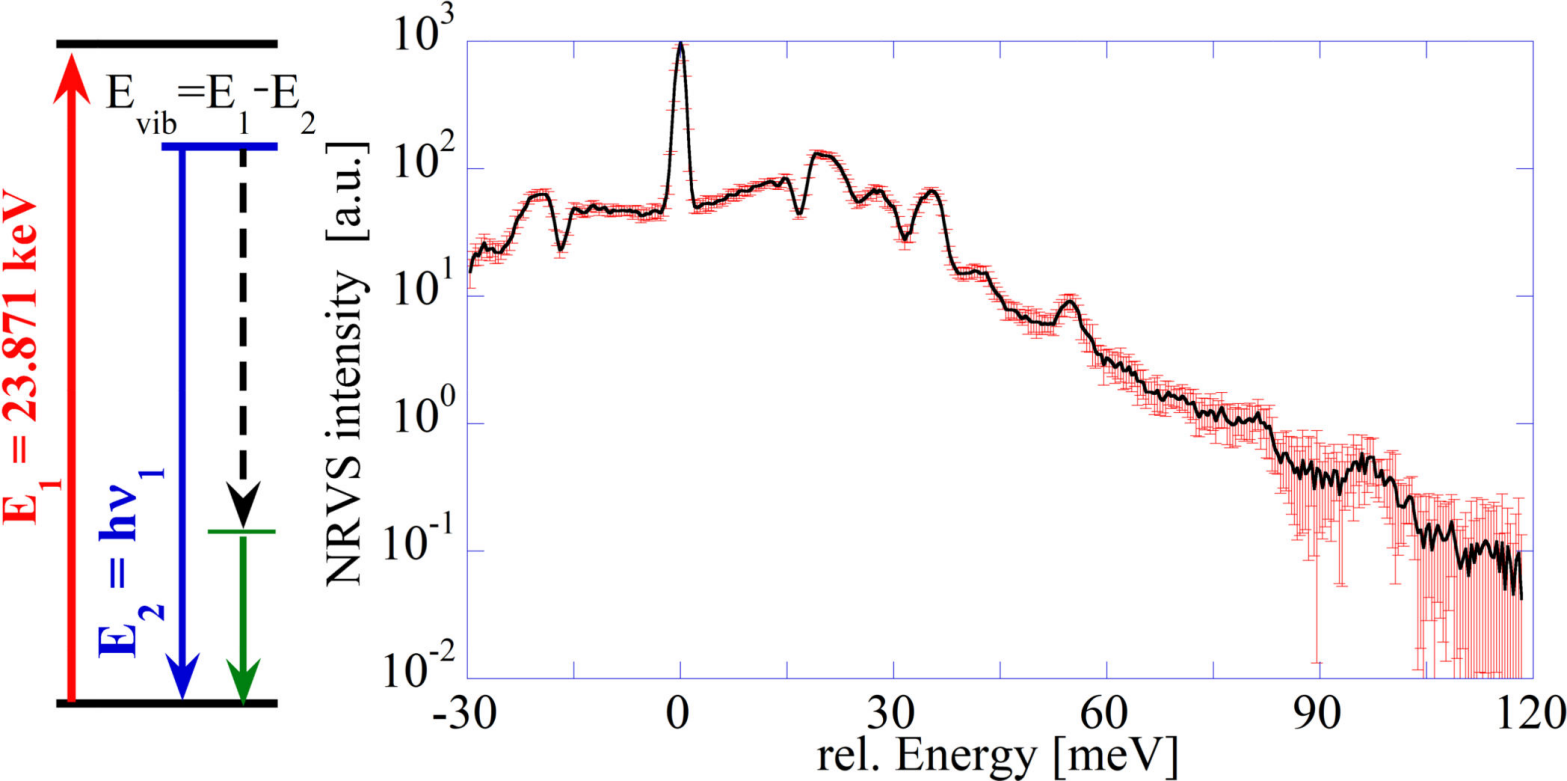
(**Left**) Grotrian–Jablonski diagram for NRVS transitions. (**Right**) NRVS raw spectrum of BSO, with an elastic line at 0 meV, Stokes lines at around 20 meV, and anti-Stokes line at around − 20 meV.

We briefly explain how an NRVS spectrum is generated [8,24]. The BaSnO3 pellet is scanned with an X-ray beam of varying energy in the range covering the nuclear γ-transition at E1 = 23.871 keV of ^119^Sn and the associated vibrational levels. This will cause a nuclear back radiation as scattered energy E_2_ = hν_1_, as shown in the scheme on the left of Figure 1, which can be recorded. In addition, fluorescence from K shell electrons due to internal conversion is observed at energy hν_2_. Both types of intensity at hv1 and hv2 are recorded versus the vibration energy E_vib_ = E_1_ − E_2_ = 23.871 keV − hν_1_. These processes can be summarized in a Grotrian–Jablonski diagram (compare [26]). This spectrum resembles an optical Raman spectrum with an elastic peak along with Stokes and anti-Stokes shifted peaks. As the method exploits the effect of nuclear transitions, it has a relation to Mössbauer spectroscopy. The NRVS absorption of γ-rays is a nuclear resonant inelastic scattering process accompanied by the creation and annihilation of phonons. The ratio of the recoil-free nuclear resonance absorption to the total absorption of a material is known as the Lamb–Mössbauer factor (f_LM_).

The Lamb–Mössbauer factor for ^119^Sn (Sn4+ in BaSnO3) obtained after refinement of the spectra was f_LM_ = 0.7268 ± 0.0025, which is gratifyingly large in comparison to 0.4 for Sn^2+^ and 0.04 for Sn^0^. For comparison, the f_LM_ of SnO_2_ was reported to be as high as 0.57 in Ref. [27] and 0.628 under ambient conditions in Ref. [28].

### 2.4. Nuclear Magnetic Resonance Spectroscopy (NMR)

Prior to the NMR measurements, defined quantities of tin metal were dissolved in weighed quantities of 37% hydrochloric acid. We observed the complete dissolution of tin after 3 h at the latest under slight hydrogen development.

^119^Sn (^117^Sn) NMR experiments on dissolved tin metals were performed at 149.2 (142.5) MHz using an Avance III 400 MHz NMR system (Bruker Biospin AG, Fällanden, Swioerland). A 5 mm CryoProbe^™^ Prodigy probe equipped with z-gradient was used to perform single-pulse NMR experiments, with 90° pulse lengths of 13.1 (15.0) μs. Recycle delays of 32 s enabled quantitative recording of the spectra, and the samples were usually prepared in such a concentrated manner that signals could be obtained after just a few scans, and a reliable signal-to-noise ratio was achieved.

**Figure 2.**
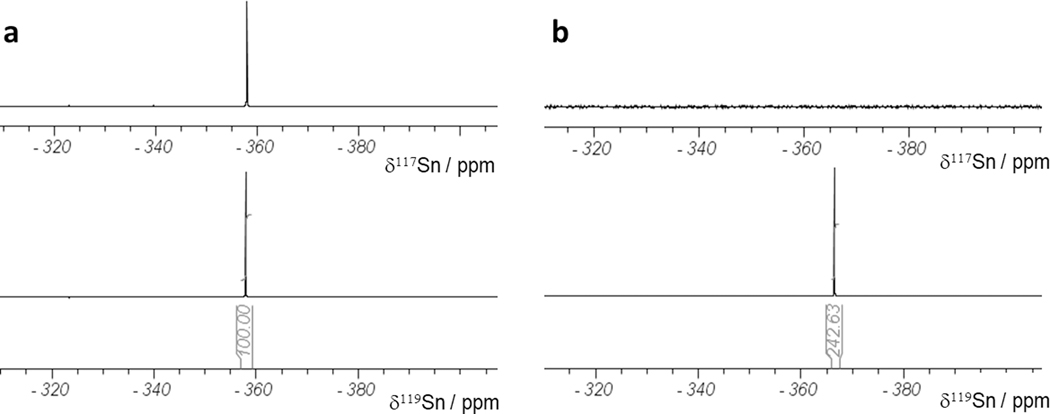
^117^Sn and ^119^Sn NMR spectra with integrals of HCl solutions of (**a**) tin metal with a natural isotope distribution of 8.59% of ^119^Sn and (**b**) ^119^Sn isotope–enriched material (the ^117^Sn NMR spectrum has been recorded with 5120 scans showing no signal at all).

In Figure 2a, the ^117^Sn and ^119^Sn NMR spectra obtained for 163.57 mg of tin metal with natural isotope distribution dissolved in 2130.0 mg of 37% HCl solution were both obtained with 16 scans only. In Figure 2b, 6.56 mg of isotopically enriched tin material was dissolved in 686.6 mg of 37% HCl solution. The ^119^Sn NMR spectrum recorded with 32 scans shows a clear resonance (with the integral calibrated relative to the integral of the sample with natural Sn abundance), while in the 117Sn NMR spectrum recorded with 5120 scans, no signal is observed. According to Equation (1), the isotopic enrichment can be calculated as follows:

(1) $${119_{Sn(\%)}}_{enr}={119_{Sn\%}}_{nat}\cdot \frac{m_{nat}}{m_{enr}}\cdot \frac{m(LM{)}_{enr}}{m(LM{)}_{nat}}\cdot \frac{NS_{nat}}{NS_{enr}}\cdot \frac{Int_{enr}}{Int_{nat}}$$

where “nat” means Sn metal with natural isotopic distribution, “enr” means the ^119^Snenriched Sn foil, ^119^Sn(%) is the isotopic enrichment, m = weight of tin metal before dissolution in HCL solution, m(LM) = weight of added HCl solution, NS = number of scans recorded for the ^119^Sn NMR spectrum, and Int = integral value of ^119^Sn NMR signal. After inserting all the values given in the text or the integrals from Figure 2, the ^119^Sn content of the enriched material is calculated to be 84%.

### 2.5. Theoretical Calculation of Total and Partial Vibrational Density of States (PVDOS)

The total and partial vibrational density of states (PVDOS) of BaSnO_3_ were calculated with respect to density functional theory (DFT) using the Quantum ESPRESSO package. For comparison, the electronic structure and phonon structure of BaSnO_3_ has been previously calculated, for example, by Bog, Jo, and Cheong [29]. We carried out our density functional theory (DFT) calculations with the Quantum ESPRESSO package [30–32] with generalized gradient approximation (GGA), as parametrized by Perdew, Burke, and Ernzerhof (PBE functional) [33] with Hubbard U correction, which is an on-site Coulomb interaction parameter used for the treatment of the electron self-interaction. Core electrons were treated with projector-augmented wave pseudopotentials available in the standard solid-state pseudopotentials (SSSPs) library (http://materialscloud.org/sssp, accessed on 1^st^ May 2025) [34]. The neutron diffraction results of BaSnO_3_ were used for all the calculations; a = 4.11 Å in a cubic structure with the Pm30m space group. A Hubbard U term of 8 eV was applied to O 2p electrons. In all calculations, the cutoff energy was 80 Ry for the kinetic energy and 600 Ry for the charge density. Brillouin zone integration was performed with Gaussian spreading with 0.01 Ry.

Phonon structures were calculated with the finite displacement method using the PHONOPY package [35,36]. Calculations were performed with the same parameters using an 8 × 8 × 8 Monkhorst–Pack shifted grid of k-points. Phonon properties were calculated using a 3 × 3 × 3 supercell with 270 atoms in total. The non-analytical term correction was performed with standard PHONOPY implementation.

To fit the calculated Sn-projected phonon density of states to the experimental NRVS data, the Hubbard U parameter and external strain applied to the cell were tuned manually. The optimized parameters were 1% compressive strain and a Hubbard U parameter of 8 eV. The Hubbard parameter used was validated by a perfect match with the experimental band gap [37]. We justify the application of strain in the simulation cell by the presence of vacancies in both Sn and O positions in the real material, as observed by neutron diffraction, which leads to the contraction of the unit cell [38]. We recall the relevance of vacancies to transport properties [20].

## 3. Results

### 3.1. Crystallographic Structure

**Figure 3.**
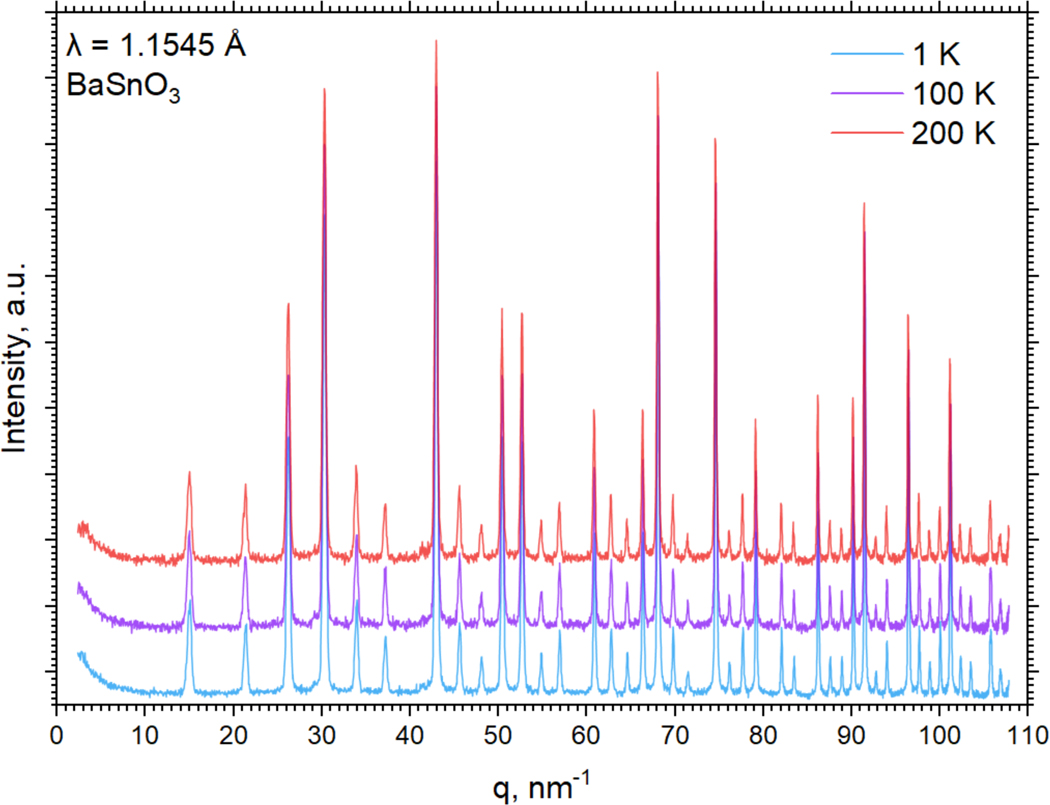
Neutron diffraction patterns of BaSnO_3_ measured at temperatures of 1, 100, and 200 K. The patterns are vertically offset.

The high-resolution neutron diffraction patterns of BaSnO_3_ shown in Figure 3 confirm that the sample is in a cubic single-phase perovskite with the space group of $\mathrm{Pm}\overset{-}{3}m$.

The lattice parameters determined by Rietveld refinement (see Table 1) agree with those reported in the literature [39]. Small deviations from the ideal structure are commonly observed for this material and may originate from minor oxygen or tin deficiency (see, for example, [40–42]). As the temperature increases from 1 to 200 K, all thermal factors—U_iso_ (Ba, Sn) and U_11_, U_33_ (O)—increase, which results in a slight expansion of the unit cell of the sample. However, the crystallographic phase is not affected by the increase in temperature.

### 3.2. Experimental and Calculated PVDOS

To understand the NRVS spectra, we recall that we excited the sample with an X-ray energy of 23.871 keV, which is the γ-transition energy of the ^119^Sn isotope [23]. Therefore, the nuclear, inelastic scattered intensity is resonant and thus specific to Sn. Therefore, the spectrum represents only the partial vibrational density of states (PVDOS) of the Sn atoms in the BaSnO_3_.

**Figure 4.**
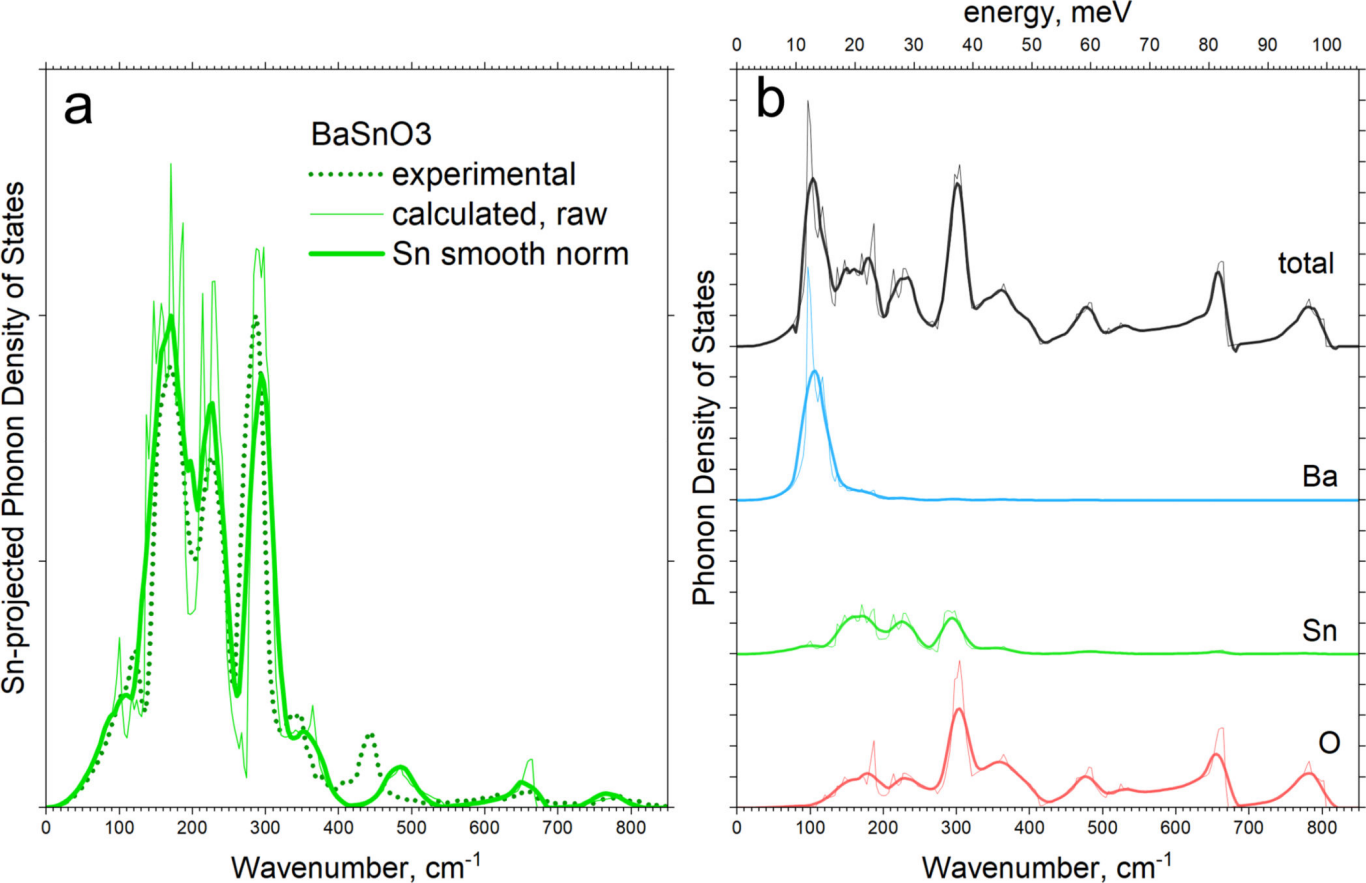
(**a**) Comparison of the experimental PVDOS derived from ^119^Sn NRVS, with the calculated PVDOS. (**b**) Sequence of calculated element-projected and total vibrational density of states (PVDOS) of BaSnO_3_: thin line—raw calculated PVDOS; thick line—calculated spectra with applied Gaussian smoothing. For better comparison, the spectra are shifted on the abscissa.

Figure 4 compares the experimental ^119^Sn-projected PVDOS with the computed PVDOS. The experimental spectrum is shown by the green dotted curve. Prominent high-intensity peaks extend over the range from 150 to 350 wavenumbers. Low-intensity peaks are distributed over the range from 350 to 800 wavenumbers. The calculated PVDOS is shown by solid lines. It is obvious that all prominent peaks determined with NRVS overlap well with the calculated PVDOS.

The right panel shows the calculated and element-projected PVDOS for oxygen, tin, and barium, as well as the total PVDOS. Given that at least one of the three components of the PVDOS, the Sn spectrum, is available as an empirical spectrum, we can work with the computational and also the conventional Raman spectroscopy-based PVDOS and the PVDOS obtained by inelastic neutron scattering with higher confidence. The spectroscopic assignment of the prominent peaks is summarized in Table 2. The two left columns list the peak positions of the experimental and calculated PVDOS in this work. They mostly align well with those from Stanislavchuk et al. [43] in the middle column. The two right columns denote the corresponding modes, as reported in [43,44].

## 4. Discussion

**Figure 5.**
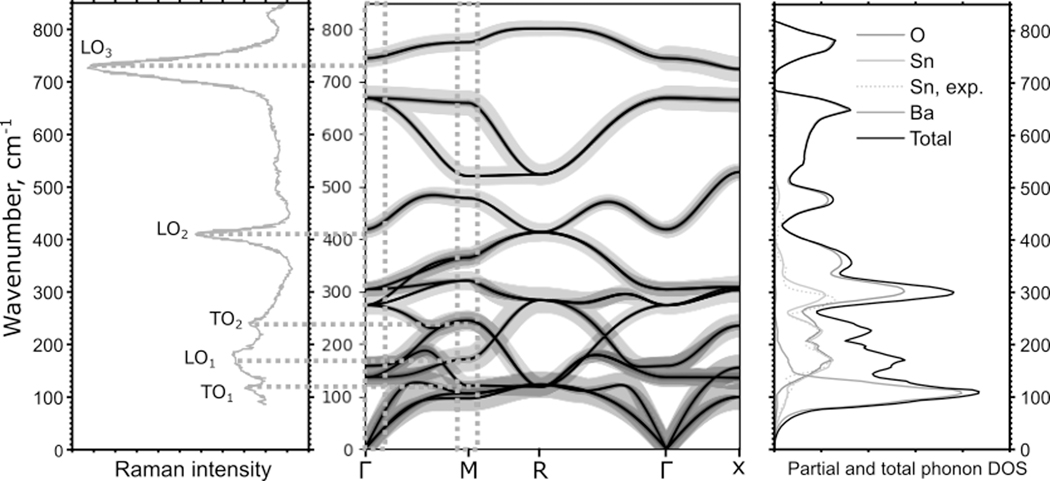
Left: optical Raman spectrum of BaSnO_3_, reproduced from [43] by digital tracing [45]; center: calculated phonon dispersion curves. The color and thickness of the lines correspond to the contribution of the corresponding atom type to the phonon mode; right: calculated element-projected phonon DOS and experimental Sn-projected PDOS, derived from the NRVS spectrum.

In Figure 5, we summarize the comparison between the information available from the optical Raman spectroscopy and NRVS. Conventional Raman spectroscopy shows phonon modes around the Г-point of the Brillouin zone (or M-point, if the symmetry of the unit cell is lowered [43]) with a low value of q; i.e., it detects only a small fraction of the full phonon dispersion structure, as shown with orange dotted rectangles in Figure 5.

The experimental Raman spectrum is in good agreement with the calculated phonon structure, showing distinct optical longitudinal vibration modes, LO_2_ and LO_3_, corresponding to the modes at the Γ point, as well as broad transversal optical TO_1_, LO_1_, and TO_2_ peaks, corresponding to phonon modes at the Γ points or M points of the Brillouin zone, which become visible since the M point becomes the center of Brillouin zone when the symmetry lowers from the Pm-3m to the Pnma space group [43].

The phonon structure and dispersion curves were in turn obtained by fitting the calculations to the NRVS spectra. Therefore, our results demonstrate agreement between the synchrotron X-ray-derived NRVS and conventional optical Raman vibration spectroscopy. At the same time, NRVS provides information on the Sn-related vibrations over the entire Brillouin zone and gives a different angle and more comprehensive description of the vibration structure.

According to theories that link lattice vibrations to proton conductivity, the modes that facilitate the transport of protons are associated with the counter-motion of adjacent oxygen atoms [46], i.e., oxygen-related phonons at the edge of the Brillouin zone. As we can see in the phonon dispersion curves in Figure 5, Sn vibrations have a strong overlap with oxygen modes, making NRVS an efficient tool for studying the modes most relevant to the functional properties of the material.

Song et al. [47] calculated the phonon structure of BaSnO_3_ and assigned the phonon bands at lower frequencies to longitudinal and transversal acoustic modes, which originate predominantly from the heavy Ba and the Sn ions. While their results generally agree well with our calculations, the calculations by Song et al. did not include the non-analytical term correction, which led to the LO-TO splitting around the Г-point, particularly noticeable for modes around 400 and 700 cm^−1^. Comparison of the calculation results with experimental Raman spectra demonstrates the necessity for this correction. In general, our phonon band assignments show no contradictions with those in the references [43,47].

Close inspection of this region shows that the experimental NRVS-derived PVDOS produces a distinct peak at 440 cm^−1^ (clear in Figure 4b). The corresponding calculated peak is located at 490 cm^−1^, almost 50 cm^−1^ higher. The peak in Sn-projected PVDOS originates from the branch that produces the LO_2_ mode at the Γ point, which involves a certain contribution from both Sn and O atoms. The discrepancy between the Raman spectrum (LO_2_ mode) and the calculated energy of the branch at the Г-point is only ~10 cm^−1^, illustrating that NRVS shows the discrepancy between the real and calculated vibration structure more strongly. While the full real and element-specific vibration structure is largely inaccessible through experimental measurements, the calculated structure clearly has its limitations and inaccuracies.

It would be highly valuable if the element-projected phonon DOS could be experimentally accessible for every element and all crystallographic sites in a compound. This is currently not possible, unless the material contains one of the 16 elements listed in Figure 10.4 in the book by S.P. Cramer [48]. KTaO_3_ is a proton conductor, where the isotopes ^40^K (already demonstrated) and ^181^Ta (feasible, but not yet demonstrated) can, in principle, be probed with NRVS, and thus, two out of three components could be experimentally assessed, and only the oxygen lattice would be the missing experimental information. In the present case of BaSnO3, only the partial phonon DOS of the tin sublattice is experimentally available, but this still represents progress compared to the guesswork required when relying solely on computational phonon DOS. Additional empirical—i.e., experimental—data should increase confidence in any computations. Therefore, when the experiment demonstrates a discrepancy from the model, it provides deeper insights into the actual properties of the material. This study illustrates the strength of NRVS compared to Raman spectroscopy in providing more information on the vibrational structure of the material.

## Figures and Tables

**Table 1.** Structure parameter from Rietveld refinement for BaSnO_3_ sample at 1K, 100 K, and 200 K.

BaSnO_3_, wR = 5.11%			
T, K	1 K	100 K	200 K
a, Å	4.11016(7)	4.11113(10)	4.11329(11)
Ba occ.		1	
Sn occ.		0.970(3)	
O occ.		0.975(3)	
U_iso_(Ba), Å ^2^	0.00167(12)	0.00272(17)	0.00402(18)
U_iso_(Sn), Å ^2^	0.00068(10)	0.00120(15)	0.00184(16)
U_11_(O), Å ^2^	0.00498(11)	0.00571(17)	0.00768(19)
U_33_(O), Å ^2^	0.00228(21)	0.00299(31)	0.0033(3)

**Table 2.** Position and assignment of phonon modes of BaSnO_3_.

PVDOS Peak Position [cm^−1^]			Assignment	
This Work Exp.	This Work Calc.	Ref. [43]	Ref. [43]	Ref. [44]
105, 122	100, 120	115	TO1	Ba-SnO_3_ translation
153, 170, 185	150, 170, 187	150–170	LO1	
216, 227, 240	214, 229, 240	238	TO2	O-Sn-O bending
275, 295, 310	266, 288, 297			
334, 347	340			
409	-	408	LO2	Sn-O_3_ torsion
433, 446	426, 441 very low			
465, 487	465, 483			
655	662			
-	-	724	LO3	Sn-O stretching

## Data Availability

The data can be obtained upon personal request by contacting Artur Braun and Alexey Rulev.

## Abbreviations

The following abbreviations are used in this manuscript:

DOS: Density of States
NAC: Non-Analytical Contribution
NMR: Nuclear Magnetic Resonance
NRIXS: Nuclear Resonant Inelastic X-ray Scattering
NRVS: Nuclear Resonance Vibration Spectroscopy
PVDOS: Partial Vibration Density of States
TO: Transversal Optical
LO: Longitudinal Optical
